# The Role of Functional Amyloids in Multicellular Growth and Development of Gram-Positive Bacteria

**DOI:** 10.3390/biom7030060

**Published:** 2017-08-07

**Authors:** Anna Dragoš, Ákos T. Kovács, Dennis Claessen

**Affiliations:** 1 Terrestrial Biofilms Group, Institute of Microbiology, Friedrich Schiller University Jena, 07743 Jena, Germany; anna.dragos@uni-jena.de; 2 Department of Biotechnology and Biomedicine, Technical University of Denmark, 2800 Lyngby, Denmark; 3 Institute of Biology, Leiden University, 2333BE Leiden, The Netherlands

**Keywords:** amyloid fiber, *Streptomyces*, *Bacillus subtilis*, chaplin, TasA, biofilm, extracellular matrix, development, multicellular growth

## Abstract

Amyloid fibrils play pivotal roles in all domains of life. In bacteria, these fibrillar structures are often part of an extracellular matrix that surrounds the producing organism and thereby provides protection to harsh environmental conditions. Here, we discuss the role of amyloid fibrils in the two distant Gram-positive bacteria, *Streptomyces coelicolor* and *Bacillus subtilis*. We describe how amyloid fibrils contribute to a multitude of developmental processes in each of these systems, including multicellular growth and community development. Despite this variety of tasks, we know surprisingly little about how their assembly is organized to fulfill all these roles.

## 1. Introduction

Many proteins have the propensity to assemble, under particular conditions, into stable amyloid or amyloid-like fibrils. These fibrils have been mostly studied in the context of debilitating diseases such as type two diabetes, Alzheimer’s and Parkinson’s [1,2]. However, over the past 15 years, we have become aware that such fibrils are not only associated with diseases, but in fact occur abundantly in nature in all domains of life where they can have various useful roles [3,4,5,6]. These so-called functional amyloids are often found in the extracellular matrix of bacterial communities or on the cell surface of various microorganisms, where they generally serve protective roles. However, functional amyloid fibrils have also been identified in so-called melanosomes in human cells, where they contribute to the efficient templating and conversion of a toxic melanin precursor [7]. In contrast to the disease-causing amyloid proteins, those with functional roles appear to be assembled mostly on the outside of the cell or in contained compartments within the cell, which probably mitigates potential detrimental effects if correct assembly fails. In addition, functional amyloid assembly may involve specialized machinery that controls the polymerization process [5]. In this review, we describe how functional amyloids function and assemble in two Gram-positive bacteria, *Streptomyces coelicolor* and *Bacillus subtilis*. These proteins appear to have multiple roles during growth and development, while we know surprisingly little about how their assembly is orchestrated in space and time to achieve these diverse functions.

## 2. Functional Amyloid Proteins in *Streptomyces coelicolor*

Streptomycetes are filamentous microbes that are abundantly present in most soil environments [8]. These multicellular bacteria thrive by establishing a mycelial network of interconnected filaments, called a mycelium. This mycelium feeds on complex polymers in the soil, which are enzymatically degraded into smaller molecules. When nutrients become scarce, a development program is initiated, during which filaments escape the aqueous environment to grow into the air [9]. These so-called aerial hyphae may further develop into chains of spores that are not only better equipped to withstand the harsh conditions, but are also able to establish new colonies elsewhere. The commitment to aerial growth is accompanied by major changes in the organization of the mycelial network and properties of the hyphae. For instance, part of the vegetative mycelium undergoes massive lysis [10], which leads to the release of nutrients and building blocks that can be used to construct these new hyphae. Furthermore, in contrast to vegetative hyphae aerial hyphae are hydrophobic. This hydrophobicity is due to the assembly of an amyloidal fibrillar surface layer that envelopes the spores [11,12]. The proteins that make up this surface layer are called chaplins, for coelicolor hydrophobic aerial proteins, after the model organism *Streptomyces coelicolor* in which they were first discovered. *S. coelicolor* makes eight chaplins (ChpA-H), which can be classified into two main groups based on their size: ChpA-C are approximately 225 amino acids long, whereas ChpD-H are significantly shorter (±55 amino acids). All chaplin proteins contain one (ChpD-H) or two (ChpA-C) conserved chaplin domains (Pfam 03777 or DUF320), which in essence comprise the sequence of the mature form of the short chaplins [13]. Notably, unlike the short chaplins, ChpA-C contain a canonical C-terminal sortase signal, which is recognized by sortase enzymes that covalently couple their substrates to the peptidoglycan present in the cell wall. Indeed, direct evidence for sortase-mediated coupling of ChpC has been demonstrated, and it is expected that ChpA and ChpB are also anchored to the cell wall [12,14].

The amyloidal surface layer formed by the chaplins is highly insoluble, a feature shared with other assembled amyloidal proteins. Assembled chaplins resist boiling in the detergent sodium dodecyl sulfate (SDS), and can only be extracted from the cell surface using trifluoroacetic acid (TFA) [11]. In this manner, ChpD-H were readily isolated from the cell surface, together with the so-called rodlin proteins (see below) [11]. However, the long chaplins ChpA-C were not detected, presumably due to their covalent coupling to the peptidoglycan. In vitro experiments using mixtures of monomeric chaplins have demonstrated that these proteins have a strong propensity to self-assemble into an amphipathic membrane when confronted with a hydrophobic-hydrophilic interface [15,16]. The hydrophobic side of this membrane is characterized by small 4–6 nm-wide fibrils that are very similar in appearance to the fibrils that are observed on the surface of spores. By contrast, the hydrophilic side of the membrane is relatively smooth. Based on these in vitro findings, it was proposed that chaplins also assemble into an amphipathic surface layer at the cell-wall–air interface, whereby the hydrophilic side faces the cell wall, and the hydrophobic fibrillar side is exposed to the air. Co-assembly of the short chaplins with the chaplin domains present in the long chaplins would contribute to tethering the fibrillar layer to the cell surface. Conclusive evidence for the amyloidal nature of assembled chaplins was obtained by diffraction analysis, which revealed the presence of the canonical cross-beta structure in the fibrils [15,17]. Interestingly, such diffraction studies also indicated that not only mixtures of short chaplins were amyloidal, but also fibrils formed by the individual short chaplin proteins [17]. Until now, it is still largely unknown if the various chaplin proteins contribute differently to the formation of the amyloidal surface layer at the hyphal surface, and how the assembly process is orchestrated in space and time.

### 2.1. Chaplins Are Surfactants Mediating the Escape of Hyphae into the Air

Analysis of numerous constructed mutant strains lacking combinations of chaplin genes has demonstrated that a certain redundancy exists in functionality between the chaplins [18]. In general, the more chaplin-encoding genes were deleted, the more severe the phenotype was with respect to the ability to form aerial hyphae [19]. When all chaplin genes were deleted, aerial growth was largely abolished, and the very few aerial hyphae were lying on top of the vegetative mycelium. Apparently, surface hydrophobicity is crucial to allow hyphae to remain erect. A minimal chaplin strain indicated that in particular the short chaplins ChpE and ChpH, together with the long chaplin ChpC, are crucial for morphogenesis [18,20]. These three chaplins are also conserved in all sequenced genomes of sporulating streptomycetes, further substantiating their importance. Notably, the short chaplins ChpE and ChpH are formed well before aerial growth has started [11]. These short chaplins have a second distinct role in morphogenesis. They allow hyphae to escape the aqueous environment, by dramatically lowering the water surface tension at the medium–air interface [11]. Interestingly, this ability was observed at low protein concentrations, but was lost at very high concentrations (>500 μg·mL^−1^) [17]. At high concentrations, chaplins quickly assemble into a rigid amyloidal membrane at the water–air interface. This indicates that the ability of chaplins to lower the surface tension precedes the formation of this rigid membrane. 

How can this behavior be explained? The assembly of chaplins into an amphipatic amyloidal membrane may be a two-step process [16]. Initially, monomers accumulate at the water–air interface, leading to the formation of an intermediate, non-amyloidal membrane with high surface activity. At high protein concentrations, this intermediate surface-active state is only briefly present, and assembly into the final amyloid state proceeds quickly. At low protein concentrations, the speed of assembly into amyloids may be slower, thereby increasing the time and state of high surface activity. Circumstantial evidence for such an intermediate conformation was obtained when the behavior of chaplins was analyzed at elevated pH levels [16]. Under these conditions, high surface activity was detected, which was dependent on the presence of chaplins, and which was reversible when the pH was lowered to physiological conditions. Notably, under these conditions chaplins were shown to self-assemble into an interfacial membrane, which, unlike the membrane formed at physiological pH, had a semi-liquid nature and self-healing properties when punctured. This is markedly different from the rigid amyloidal membrane formed at physiological pH, which remained ruptured after puncturing. Taken together, these results indicate that the chaplins were assembling into a non-amyloidal membrane coinciding with a state of high surface activity. The elevated pH may thus have trapped the intermediate state in the assembly process of chaplins.

### 2.2. Functional Homologues with Chaplin-Like Functions in Streptomycetes

**Figure 1:**
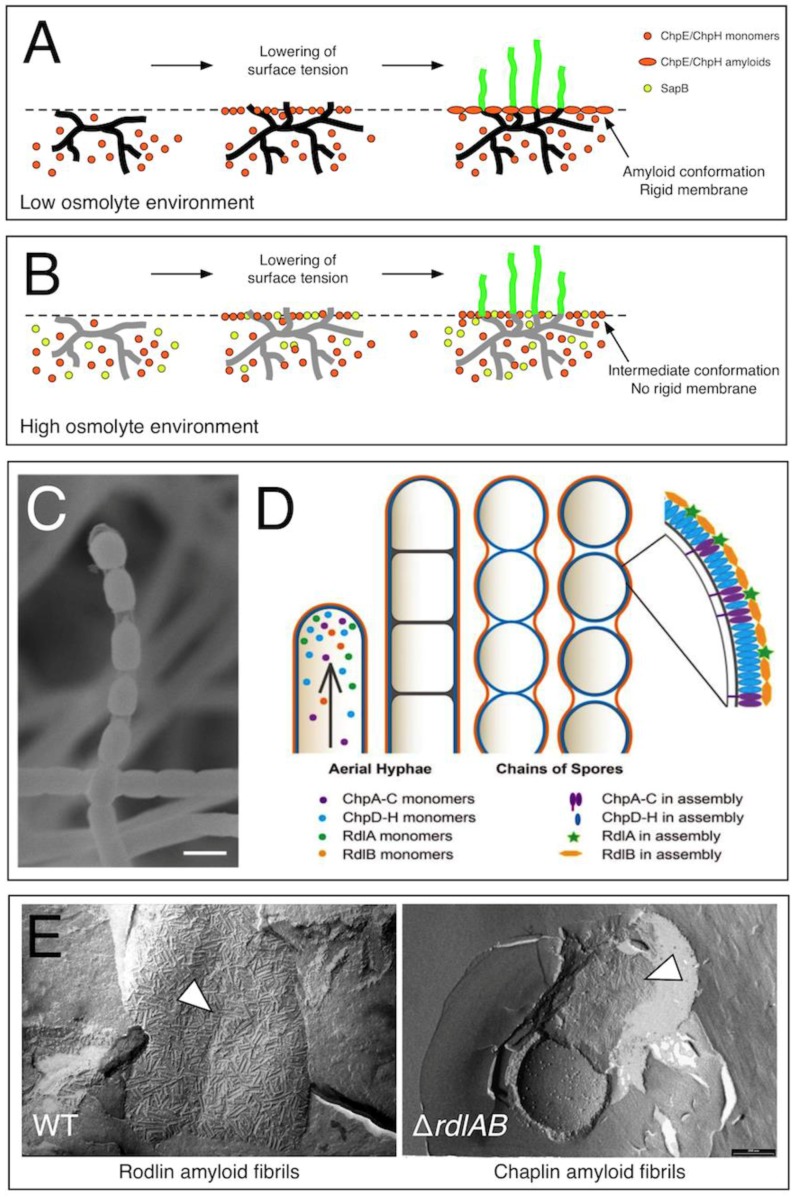
The role of chaplins and rodlins in aerial growth in *Streptomyces coelicolor*. The chaplins ChpE and ChpH are secreted by vegetative hyphae, and accumulate at the medium–air interface. Initial assembly of chaplins into a non-amyloidal membrane coincides with the lowering of the surface tension. When more chaplins accumulate at the interface, the transition to the amyloid state is induced. (**A**) In low osmolyte environments, hyphae have sufficient turgor pressure to penetrate this rigid membrane and grow into the air. (**B**) In high osmolyte-containing environments, the surfactant spore-associated protein (SapB) is produced in addition to ChpE and ChpH. The surfactant molecule SapB may intercalate into the chaplin membrane at the medium–air interface, thereby delaying or even preventing chaplins from assembling into a rigid amyloid membrane. This allows hyphae to grow into the air, even when their turgor pressure is reduced. Vegetative hyphae with a high turgor pressure are shown in black (**A**), while those with a reduced turgor pressure are shown in grey (**B**). (**C**) Visualization of spore chains using scanning electron microscopy reveals the presence of a transparent, sheath-like structure enveloping the separating spores. (**D**) This suggests that developing spore chains are surrounded by two distinct surface layers, containing assembled chaplins (purple/blue) and rodlins (green/orange). (**E**) The rodlins are part of the outermost surface layer, which has a more robust appearance (left panel) compared to the layer formed by the chaplins (right panel). Figure 1A,B was reproduced from reference [27] with permission from Springer (2014), while Figure 1C,D was reproduced from reference [28] with permission from the Nature Publishing Group (2017).

Although chaplins are instrumental for the initiation of aerial growth in streptomycetes, other molecules have been described that fulfill a similar role [21]. The best-known example is the spore-associated protein (SapB) of *S. coelicolor*, but homologous peptides have been identified in a number of other streptomycetes [22,23,24]. SapB is produced in high osmoloyte-containing environments, and was demonstrated to have surface tension-reducing activity [25,26]. Although chaplins and SapB may be partially redundant, both could also work cooperatively. In high osmolyte environments, the hyphal turgor pressure is reduced, which makes the escape of hyphae into the air more difficult. This would be particularly evident when chaplins quickly assemble into a rigid membrane at the medium–air interface, which imposes a physical barrier. Therefore, we hypothesize that SapB may facilitate aerial growth by impeding the fast assembly of the chaplin layer at the medium–air interface (Figure 1, [27]). While the presence of SapB would not prevent assembly, its physical presence would reduce the local concentration of assembling chaplins at the medium–air interface. As a consequence, the formation of a rigid membrane would be delayed, while extending the time of high surface activity (see above). This would explain why SapB is so important under conditions where hyphal turgor pressure is reduced. In conditions of low osmolarity, hyphal turgor pressure would be sufficient to allow hyphae to breach the rigid membrane formed by the chaplins at the medium–air interface. This potential cooperativity between these surfactant molecules awaits further investigation.

### 2.3. Developing Aerial Hyphae Are Surrounded by a Second Amyloidal Layer Formed by the Rodlin Proteins

Recent evidence indicates that the developing hydrophobic spores are surrounded by a second distinct surface layer, called the rodlet layer. This layer is characterized by pairwise aligned rods, that are considerably larger than the fibrils formed by the chaplin proteins. These rods are up to 450 nm in length, and have a diameter of 8–10 nm [29]. Biochemical characterization and genetic analyses indicate that this layer depends on two proteins, called rodlins (Rdl) [30]. Both proteins, RdlA and RdlB, are highly similar (>90%) and have a non-redundant function in the formation of the rodlet layer [19,28]. Deletion of either *rdlA* or *rdlB* prevented the formation of the rodlet layer.

Unexpectedly, only RdlB was able to form amyloid fibrils in vitro [28]. These fibrils were very reminiscent of the fibrils that were observed when the rodlet layer was isolated by mechanical disruption [31]. In particular, the fibrils appeared to align into pairwise bundles. Further characterization identified a 26 amino acid residue region within the N-terminus of RdlB that explained the fibril forming propensity [28]. Although the hydropathy patterns of this region were very similar between RdlA and RdlB, more charged residues were present in RdlA. Excitingly, the replacement of three key discriminating residues in RdlA was sufficient to allow this mutant protein (RdlA*) to form amyloid fibrils in vitro and in vivo. While RdlA* could be considered the first example of an engineered functional amyloid protein in vivo, little is known as to how RdlA regulates the formation of the rodlet layer.

The discovery that rodlins potentially form a second surface layer surrounding developing spore chains raises numerous questions, such as how the assembly process is coordinated with that of the chaplin proteins. It was initially proposed that both classes of proteins work cooperatively in the formation of the rodlet layer [19]. However, more recent data supports an alternative model in which both classes of proteins assemble into independent layers on the hyphal surface (Figure 1) [28]. The innermost chaplin layer would envelop the spores and provide them with surface hydrophobicity. This hydrophobicity is beneficial for efficient dispersal. The rodlins would assemble into an additional hull, which could contribute to further stabilization of the developing aerial hyphae. In addition, it may be important to ensure that spores remain associated with the colony until they have matured completely. Perhaps without the rodlins, immature spores get detached from the colony more easily, which may significantly impact their viability. This may be particularly relevant for *Streptomyces* strains that are able to generate spores in liquid environments (e.g., *Streptomyces griseus* and *Streptomyces venezuelae*), of which we know that they possess rodlets. However, this awaits further investigation.

## 3. Diverse Functions of the TasA Amyloid in *Bacillus subtilis*

*Bacillus* are widespread Gram-positive bacteria that usually thrive in the soil, plant rhizosphere or an animal host where, depending on species, they serve as commensals or pathogens. Similarly to *Streptomyces*, *Bacillus* can sporulate under nutrient-limiting conditions. Those species examined can also form highly resistant cell assemblies called biofilms where bacteria stick to each other embedded in extracellular material. 

TasA is recognized as one of the major proteinaceous components of the *B. subtilis* extracellular matrix (ECM). TasA assembles into fiber structures of 10–15 nm width and with variable lengths [32] that look similar to the curli fibers associated with *Escherichia coli* [33] or the more recently documented amyloid fibers of phenol soluble modulins (PSMs) of *Staphylococcus aureus,* when visualized using transmission electron microscopy [34]. The fibers produced by *B. subtilis* bind gold-labelled anti-TasA antibodies and are completely absent in a *tasA* mutant [32]. Similar fibers are also formed in vitro by overproduction of full-length TasA protein, and which can be disintegrated after exposure to 10% formic acid. In addition, polymerization of TasA can be prevented by the same agents that block assembly of yeast prion protein New1 [35]. Finally, unlike the *tasA* mutant, wild-type *B. subtilis* strains can be stained with classical amyloid-specific dyes including Congo Red, Coomassie Brilliant Blue and fluorescent thioflavin T [32,36,37]. In the light of these results, TasA is often considered another example of a functional bacterial amyloid.

TasA is encoded in the *tapA-sipW-tasA* operon [32] that, together with the exopolysaccharide operon *epsA-O*, is indirectly controlled by the master regulator Spo0A [38]. Initially, TasA is synthesized as a 261 amino-acid (aa) protein, of which the 27 N-terminal residues encode the signal peptide that is removed by the SipW signal peptidase, resulting in the 234-aa mature form of TasA. TapA is suggested to stick to TasA fibers, thereby playing an important role in TasA localization and polymerization [32,37,39]. In the *tapA* mutant, TasA is positioned near the cell surface in the cytosol, and only thin, disorganized fibers are released [37,39].

### 3.1. TasA Amyloid Fibers as a Key Structural Element of the B. subtilis Extracellular Matrix 

**Figure 2:**
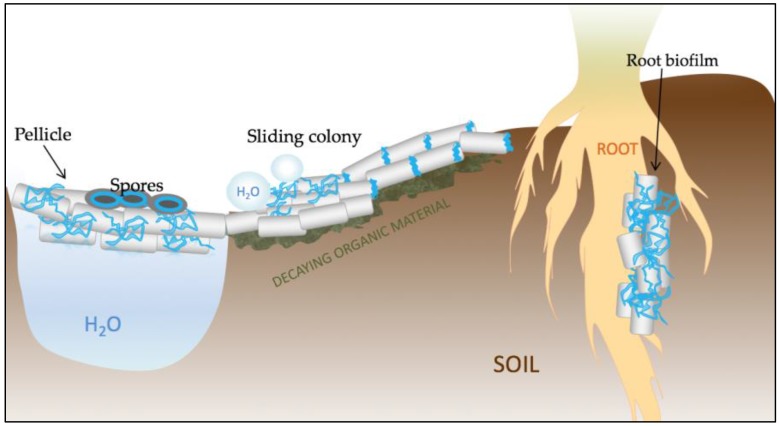
Schematic representation of the various functions of TasA in *Bacillus subtilis*. TasA (represented in blue) is a key structural element of pellicle biofilms that form at the water–air interface. The protein is also important for proper spore architecture, probably by connecting the cortex with the inner coat. TasA also contributes to maintaining the non-wetting properties of colonies and to surface spreading. Finally, TasA is crucial for the establishment of root-associated biofilms and, due to its antimicrobial properties, it may play an important role in biocontrol.

The dramatic effect of TasA fibers on bacterial fitness can be appreciated in various biofilm model systems where bacteria encounter nutrient and oxygen gradients (Figure 2). Thick biofilms formed at the liquid–air interface (so called pellicles) have become a distinguished biofilm model of *B. subtilis*. Such pellicles are not formed by mutants lacking TasA [32]. Although the lack of TasA does not constrain growth in minimal medium, it prevents *B. subtilis* from forming sticky large aggregates of cells that can float at the oxygen-rich liquid–air interface. Also, colonies formed by the *tasA* mutant exhibit reduced structural complexity as compared to the wrinkled wild-type parent [32,37,40]. Despite the key role of TasA in biofilm formation and its strong influence on colony morphology, recent biophysical analysis has revealed that *tasA* mutant colonies have a similar stiffness as the wild type [41]. Still, lack of TasA hampers the non-wetting properties of colony biofilms indicating that together with exopolysaccharide (EPS) and the hydrophobin Bacillus surface layer protein A (BslA), TasA plays an essential role in retaining biofilm hydrophobicity [42]. It was recently shown that hydrophobic colonies have dramatically increased resistance to chemical attack even independently of structural complexity [43]. Finally, *tasA* mutant strains are also defective in the formation of root-associated biofilms [44]. As the plant rhizosphere may represent the natural habitat of *B. subtilis*, TasA may be one of key secreted compounds driving the ecology and evolution of this species.

### 3.2. Role of TasA in Surface Spreading

It was recently discovered that TasA plays an important role in flagellum-independent surface spreading, called sliding, based on the discovery that *tasA* mutants fail to efficiently colonize surfaces [45]. Surface spreading depends on the formation of so called van Gogh bundles of aligned matrix-producing cells. These bundles can move forward with the help of surfactant released by a subpopulation of cells. The exact role of TasA in spreading is unclear. Although TasA is not required for bundle formation, its localization between the poles of bacterial cells suggests that TasA may influence the biophysical properties of the bundles, for example their rigidity [45]. The importance of TasA in surface spreading is further supported by the recent findings of Mhatre and collagues [40] who showed that spreading of *B. subtilis* vastly accelerates in the absence of Ca^2+^. A lack of TasA (as well as other matrix compounds like EPS or BslA) renders *B. subtilis* insensitive to the effects of calcium. Recently, the amyloid-forming PSM of *S. aureus* were identified to promote surface spreading [46]. Although microbial amyloids in general are known to promote surface adhesion rather than spreading, the first function likely determines the latter. When lysozyme-derived amyloid was used as a cell culture platform for fibroblasts, surface spreading significantly improved, most likely through efficient integrin–amyloid interactions [47].

### 3.3. TasA as a Weapon 

Antimicrobial effects of amyloids are not uncommon [48,49], and TasA also shows broad-spectrum antimicrobial properties when overexpressed in *E. coli* [50]. The expression of *tasA* in *B. subtilis* biofilms was recently shown to increase in the presence of the pathogen *Fusarium culmorum* [51] suggesting that *B. subtilis* may competitively respond to certain cues released by the fungus, however a possible antifungal effect of TasA is unknown. Interestingly, TasA also plays a role in combinatorial kin recognition between closely related *B. subtilis* strains [52,53]. Stefanic and colleagues observed that the swarms of non-kin strains avoid each other through formation of a boundary line [53]. The deletion of *tasA* prominently expands the boundary formed between the tasA mutant and other *B. subtilis* strains [52]. Therefore, TasA plays an important role in the process of kin recognition, which is probably mediated via various inhibitory molecules. It remains unclear whether this boundary between swarming bacteria is modulated by antimicrobial properties of TasA, surface spreading or simply by differences in ECM composition or structure.

### 3.4. Role of TasA in Sporulation

Although TasA gained popularity as a key ECM component in a *B. subtilis* biofilm, it was initially identified as an abundant 31-kDa component of mature spores. [50,54]. TasA is a unique coat-associated protein that is already present in the cytoplasm of predivisional cells. During sporulation, TasA is probably present in the septal lumen. After engulfment, TasA may facilitate construction of the inner coat, possibly from the septum-proximal side of the developing spore. Removal of the signal peptide by the signal peptidase W (SipW) is necessary for the proper localization of TasA [50]. The absence of TasA results in asymmetric spores that accumulate misassembled material at one pole and in an expanded inner coat and altered outer coat structure [54]. Interestingly, the lack of TasA does not affect sporulation frequency or spore resistance [50,54] and only slightly affects germination [50]. Recently, detailed analysis of the architecture of *B. subtilis* spores was revisited using atomic force microscopy [55]. The authors noticed that when the outer coat is removed from properly assembled spores using chemical treatment, rodlet structures were evident, which potentially could be amyloids. If these rodlets are formed by TasA, these observations would be in accordance with the previously proposed role of this protein supporting the cortex–inner-coat interaction [54].

### 3.5. Conserved and Less Conserved Features of TasA 

TasA homologs are widespread in other Bacilli (e.g., *B. amyloliquefaciens*, *B. velezensis*, *B. licheniformis*, and *B. pumilus*) and even in *Streptococcus pneumoniae*. For example, food-poisoning *B. cereus* encodes two orthologs of *tasA* (denoted as *tasA* and *calY*), but in contrast to *B. subtilis*, *sipW-tasA* form one operon and *calY* is separated from *sipW-tasA* by an unknown gene, and expressed independently. Similarly to *B. subtilis*, electron microscopy visualization of *B. cereus* reveals cell-surface-associated fibers during biofilm formation [56]. The fibers are no longer formed in *sipW-to-calY* or in *sipW* mutants, but *tasA* rather than *calY* seems to be essential for fiber formation. Based on the thickness of the submerged biofilms observed for different *B. cereus* mutant strains, it was proposed that CalY and TasA play roles at early (24 h) and late (72 h) stages of biofilm development, respectively. It remains enigmatic why *B. subtilis* requires an accessory protein (TapA) for fiber formation while *tapA* is absent in *B. cereus*. Introduction of the *sipW-to-calY* region of *B. cereus* into *B. subtilis* lacking the native *tapA-sipW-tasA* operon restores wrinkled pellicle formation, apparently independently of TapA [56].

Major differences in TapA are also found in *B. pumilus*, where the protein is significantly shorter than in *B. subtilis* (175 vs. 253 amino acids), with the main dissimilarities being in the C- and N-terminal regions [57]. While the C-terminal residues that are lacking in *B. pumilus* are not required for fiber formation in *B. subtilis* [37], the 8 aa region (50–57) of TapA, essential for TasA fiber formation, is not conserved in *B. pumilus*. This suggests a different anchoring mechanism of TasA by TapA or an entirely different role for TapA in matrix assembly [57].

One of the conserved features of TasA is the absence of cysteine residues. This contrasts with several other bacterial amyloids, like curli, where cysteines are not only conserved but also play an important role in fiber formation [58]. Cysteines are also present in the Fap amyloids of *Pseudomonas* spp., either in the C-terminal CxxC motif, or as a single cysteine at the 25th position of the mature protein [59]. The recently described amyloid major sheath protein A (MspA) from the methanogenic archaea *Methanosaeta thermophile* contains three conserved cysteines in non-core regions of the fibrils [6]. Still, the effects of substitution to cysteine in amyloid proteins are difficult to predict: occasionally, introduction of cysteine inhibits fibrils formation [60], while in other cases, it accelerates amyloidogenesis [61]. Two cysteine residues are also present in all chaplin proteins, except for ChpE. These cysteine residues were suggested to form an intramolecular disulfide bond. Replacement of the Cys residues in ChpH unambiguously demonstrated the importance of these residues for morphological development [18]. Interestingly, TapA, which mediates the assembly of TasA, carries five conserved cysteine residues and the substitution of three or more cysteines to alanine has a slight effect on biofilm morphology [37]. Very recently, a crucial role of cysteines was revealed for another ECM component of *B. subtilis*, called BslA, that modulates biofilm development and assures its hydrophobicity. While monomeric BslA is sufficient to maintain proper biofilm morphology, only the cysteine-bridged oligomers of BslA can render the biofilms non-wetting [43]. The importance of cysteines in BslA (or in TapA) could potentially explain their absence in amyloidogenic TasA. Since BslA and TasA coexist in the ECM, the oligomerization of the first could be disturbed by cysteine-containing TasA. Therefore, evolution of functional cysteines may be limited to one ECM component, in this case BslA. Alternatively, it was proposed that the lack of cysteines in an amyloid can ensure its higher flexibility and multi-functionality—as in case of harpins (amyloids produced by plant pathogens) [62].

TasA protein has been consistently addressed in every review manuscript on bacterial amyloids published after 2010 [36,63,64,65]. Nevertheless, in contrast to other well-established bacterial or archaeal amyloids [6,66,67,68,69], X-ray diffraction spectra of TasA, confirming the classical cross-beta type amyloid fold, have never been obtained. This could explain the precaution of authors frequently referring to TasA as an amyloid-like rather than an amyloid protein (for example in: [35,37,56,64,70]). Recently, a groundbreaking study revealed a unique cross-alpha amyloid structure of staphylococcal PSMα3 [71]. The discovery not only questions the canonical definition of a true amyloid fiber but also indicates that drawing a line between amyloid and amyloid-like fibers may be more difficult than previously anticipated.

## 4. Conclusions

Over the past 15 years we have become aware that amyloid proteins have critical functions in all domains of life. In this review, we have focused on two Gram-positive bacterial species, both of which exploit amyloid proteins for diverse functions during their life. However, when compared to the well-studied machinery underlying curli biogenesis, we know surprisingly little how these Gram-positive bacteria coordinate the process of amyloid assembly. In fact, we do not even know whether this process actually requires strict coordination. The curli biogenesis machinery, which operates in the outer membrane, is critical for translocating the amyloid forming Curlin sigma S-dependent growth A (CsgA) protein from the periplasm to the outside of the cell [5]. Given that Gram-positive bacteria lack an outer membrane, perhaps secretion of the amyloid forming proteins in the extracellular environment is sufficient to prevent toxic accumulation inside the cells. At sufficiently high concentrations on the outside of the cells, self-assembly may lead to fibril formation, following rules dictated by biophysical principles rather than those inflicted by elegant machinery.

Since functional amyloids are beneficial extracellular compounds, they could potentially be exploited by non-producing mutants or other species that thrive in the neighborhood. In the case of ChpA-C, the dilemma is probably solved by anchoring of the proteins in the peptidoglycan of the producers resulting in the privatization of the extracellular goods. On the contrary, the short chaplins ChpE and ChpH with biosurfactant properties, are secreted well before aerial growth has started [11]. Potential non-producers could therefore take advantage of ChpE and ChpH released by their neighbors, while at the same time reducing the metabolic costs associated with their synthesis. Still, the surfactant properties are observed at low protein concentrations. Therefore, the spread of potential “cheats” may simply be prevented due to the relatively low metabolic cost associated with the production of ChpE and ChpH. Alternatively, as in higher concentrations the ChpE and ChpH are able to assemble into proper amyloids, they may provide a yet unknown benefit to the producing hyphae.

To which extent is TasA shared within a *B. subtilis* population? The complementation assay involving *tasA* and *eps* mutants suggests that TasA can be shared with non-producer cells since the two strains can exchange matrix components and form a wild type-like biofilm [39,72,73]. However, in pellicles the non-producers can be in direct physical contact with the producers, which would facilitate their access to cell-associated goods. In fact, previous studies indicate that TasA remains cell-associated even in a TasA-overexpressing strain [32]. Moreover, visualization of TasA-mCherry in a spreading colony confirms that the protein stays in close proximity to the producing cells, suggesting that its diffusion is rather limited. Interestingly, while the lack of TasA reduces colony expansion, expansion cannot be improved by TasA overproduction [45]. Therefore, this might indicate that TasA acts locally and the native production level of TasA is sufficient for maximal impact. The functional diversity of amyloids can place those compounds in diverse social contexts. Next to the mechanisms of biogenesis, the evolutionary stability of functional amyloids in the light of their different biological roles, awaits further study.
